# Leakage and Thermal Reliability Optimization of Stacked Nanosheet Field-Effect Transistors with SiC Layers

**DOI:** 10.3390/mi15040424

**Published:** 2024-03-22

**Authors:** Cong Li, Yali Shao, Fengyu Kuang, Fang Liu, Yunqi Wang, Xiaoming Li, Yiqi Zhuang

**Affiliations:** 1 School of Microelectronics, Xidian University, Xi’an 710071, China; 2 Beijing Smartchip Microelectronics Technology Company Limited, Beijing 100089, China

**Keywords:** gate-all-around (GAA), band-to-band tunneling (BTBT), reliability, self-heating effect (SHE), nanosheet field-effect transistor (NSFET)

## Abstract

In this work, we propose a SiC-NSFET structure that uses a PTS scheme only under the gate, with SiC layers under the source and drain, to improve the leakage current and thermal reliability. Punch-through stopper (PTS) doping is widely used to suppress the leakage current, but aggressively high PTS doping will cause additional band-to-band (BTBT) current. Therefore, the bottom oxide isolation nanosheet field-effect transistor (BOX-NSFET) can further reduce the leakage current and become an alternative to conventional structures with PTS. However, thermal reliability issues, like bias temperature instability (BTI), hot carrier injection (HCI), and time-dependent dielectric breakdown (TDDB), induced by the self-heating effect (SHE) of BOX-NSFET, become more profound due to the lower thermal conductivity of ${\mathrm{SiO}}_{2}$ than silicon. Moreover, the bottom oxide will reduce the stress along the channel due to the challenges associated with growing high-quality SiGe material on ${\mathrm{SiO}}_{2}$. Therefore, this method faces difficulties in enhancing the mobility of p-type devices. The comprehensive TCAD simulation results show that SiC-NSFET significantly suppresses the substrate leakage current compared to the conventional structure with PTS. In addition, compared to the BOX-NSFET, the stress reduction caused by the bottom oxide is avoided, and the SHE is mitigated. This work provides significant design guidelines for leakage and thermal reliability optimization of next-generation advanced nodes.

## 1. Introduction

In recent decades, multi-gate devices have been considered the most promising devices for advanced nodes at 22 nm and beyond, with significant improvements in short-channel effects (SCEs) [1]. Compared to traditional planar MOSFETs, FinFETs exhibit higher driving capability and superior gate control ability, leading to their successful development for high-volume integrated circuits from the 22 nm to 5 nm nodes [2,3]. However, as device sizes scale down to 3 nm and beyond, FinFET faces severe SCEs due to the reduced flexibility of the fins, resulting in challenges to conventional scaling rules. Therefore, a more efficient channel geometry suppressing the SCE from all directions is critical [4,5]. Gate-all-around transistors have been widely studied due to their enhanced gate control capability with the channel surrounded by the gate. Among them, stacked nanosheet field-effect transistors (NSFETs) are regarded as promising candidates to replace FinFET technology thanks to their excellent gate control capabilities, superior current drive capabilities, variable channel widths, and FinFET-compatible processes [6,7,8,9].

Although NSFET devices exhibit excellent performance, research to optimize the performance of such devices continues. On the one hand, as the device size shrinks, the parasitic channel influence in NSFET on the leakage current becomes increasingly significant [10,11]. To address this issue, several improved solutions have been proposed. One widely implemented strategy is the introduction of punch-through stopper (PTS) doping to suppress the impact of the parasitic channel [12]. However, aggressively high PTS doping will increase the band-to-band tunneling (BTBT) current from the drain to the substrate, leading to increased leakage current and static power consumption [13,14]. In addition, the leakage current between the source and drain can be minimized by utilizing bottom dielectric isolation (BDI) on the substrate [15,16]. To balance the mobility difference between n-type and p-type devices in complementary metal oxide semiconductor (CMOS) technology, a high-quality source/drain is essential for applying strain to the channel. However, the bottom oxide will reduce the stress along the channel due to the challenges associated with growing high-quality SiGe material on ${\mathrm{SiO}}_{2}$ [17]. Therefore, the bottom oxide isolation nanosheet field-effect transistor (BOX-NSFET) has difficulty boosting the mobility of p-type devices.

On the other hand, the channel of NSFET is surrounded by gate oxide, which makes it difficult to dissipate the heat generated in the channel, thereby increasing the lattice temperature [18,19]. The rising lattice temperature will aggravate the reliability issues, such as the bias temperature instability (BTI) effect, time-dependent dielectric breakdown (TDDB), and hot carrier injection (HCI) effect [20,21]. Therefore, improving the self-heating effect (SHE) in NSFETs is also a significant focus of current research [20,22,23]. The bottom oxide isolation structure mentioned above makes it difficult for heat to transfer from the channel to the substrate due to the lower thermal conductivity of ${\mathrm{SiO}}_{2}$ compared to silicon, leading to a more profound SHE in NSFETs than conventional devices [24,25]. To alleviate SHE, one approach is to reduce the thickness of the bottom oxide isolation layer. However, achieving thin oxide isolation layers at the nanoscale is challenging, and a thin oxide isolation layer is less effective in suppressing leakage current. Alternatively, a new structure that utilizes material with higher thermal conductivity, such as diamond, can be adopted [26]. Using diamond layers under S/D regions can significantly mitigate the SHE. However, due to the significant lattice mismatch between silicon and diamond, growing diamond on silicon poses challenges.

This work proposes a novel nanosheet transistor structure that introduces a PTS doping scheme under the gate and SiC layers under S/D regions (SiC-NSFET). Compared to the NSFET with a conventional PTS scheme, implementing the PTS doping scheme only under the gate reduces the increased BTBT current caused by the aggressively high PTS doping. In addition, the SiC material is a wide-bandgap semiconductor material, which further suppresses the substrate leakage current thanks to a larger tunneling barrier width for carriers [27]. Compared to the BOX-NSFET, the SiC-NSFET can avoid stress reduction along the channel direction caused by the bottom oxide isolation layer. Moreover, due to the higher thermal conductivity of SiC layers than ${\mathrm{SiO}}_{2}$, heat generated within the channel can be transferred more efficiently from the bottom of the S/D regions to the substrate. This process helps to reduce the performance degradation caused by the SHE. Therefore, the SiC-NSFET structure can address the increased BTBT current resulting from the aggressively high PTS doping and effectively improve thermal reliability by optimizing the heat conduction path without stress reduction along the channel direction. This significant development offers valuable insights for further scaling of device sizes, particularly optimizing the substrate structure.

The rest of this article is organized as follows. Section 2 mainly introduces the device structure and electrical parameters in the simulation. The process flow and the simulation setting are also discussed. Section 3 analyzes the leakage current and the thermal reliability in NSFETs. Finally, the conclusion is presented in Section 4.

## 2. Device Structure and Simulation Methodology

### 2.1. Device Structure

The three-stacked 7-nm NSFET structures with the punch-through stopper (PTS) doping scheme used in this work are referred to in [28]. All the essential device geometry parameters are listed in Table 1.

**Figure 1:**
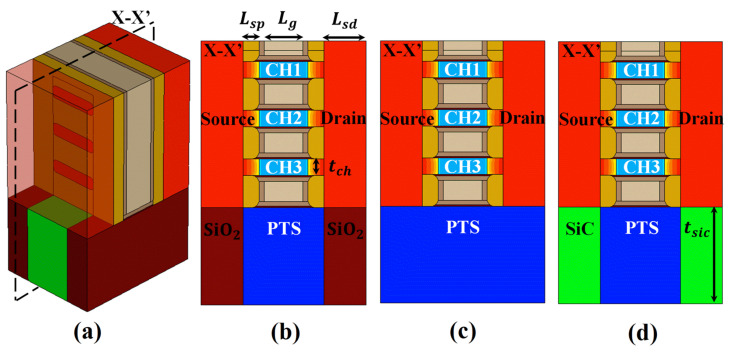
The device structures used for comparison; (**a**) 3D view of the proposed SiC-NSFET, (**b**) X-$X^{\prime }$ view of the BOX-NSFET, (**c**) X-$X^{\prime }$ view of the NSFET with a conventional PTS structure, and (**d**) X-$X^{\prime }$ view of the SiC-NSFET.

Figure 1 presents the SiC-NSFET proposed in this work, BOX-NSFET, and a conventional PTS structure. We designed a similar structure based on the cross-sectional shapes provided in [8]. The gate length ($L_{g}$) and inner spacer length ($L_{sp}$) are set to 12 and 5 nm, respectively. The S/D length ($L_{sd}$) is adjusted to 13 nm. The vertical channel space ($N_{ch}$) and channel thickness ($t_{ch}$) are set to 10 and 5 nm, respectively. For SiC layers under the S/D regions, we used the 4H-SiC during simulation, and the thickness ($t_{SiC}$) is fixed at 30 nm. The S/D regions and channels are uniformly doped, but the S/D extension regions are doped with a Gaussian doping profile. The doping concentrations of the channel and S/D regions are 1 × $10^{17}$ ${\mathrm{cm}}^{-3}$ and 1 × $10^{20}$ ${\mathrm{cm}}^{-3}$ [14], respectively. For the PTS structure, the doping concentration is set to 5 × $10^{18}$ ${\mathrm{cm}}^{-3}$ [22]. An effective-oxide-thickness (EOT) of 0.7 nm (0.45 nm of ${\mathrm{SiO}}_{2}$ and 1.5 nm of ${\mathrm{HfO}}_{2}$) is achieved. The S/D contact resistances of the NSFET are 1 × $10^{-9}$Ω·${\mathrm{cm}}^{2}$ [28].

### 2.2. Process Flow

**Figure 2:**
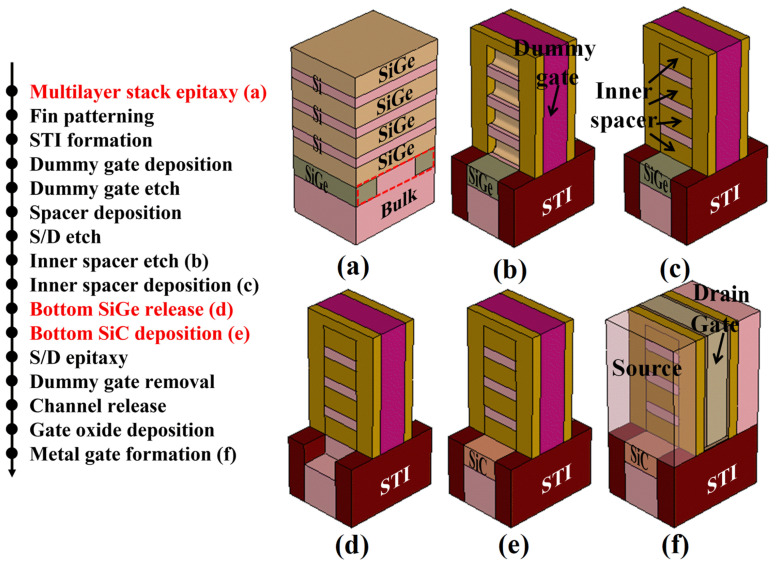
Possible process flow for the proposed SiC-NSFET. (**a**–**f**) Images illustrate the critical steps. Additional steps not used for conventional NSFETs are marked in red. (**a**) Multilayer stack epitaxy. (**d**) Bottom SiGe release. (**e**) Bottom SiC deposition.

Figure 2 shows the possible process flow for the proposed SiC-NSFET structure; the specific fabrication steps are referred to [15]. The process sequence for the SiC-NSFET structure is as follows.

The proposed SiC-NSFET can be fabricated on the bulk silicon substrate. First, to implement SiC layers under S/D regions, the substrate is etched to form a sub-fin instead of obtaining an additional S/D recess during the subsequent S/D etching process. The benefit of this is that highly uniform recess patterns can be obtained, which reduces the variations of the SiC layer.

Then, bottom thick SiGe layers containing a high % of Ge are grown on both sides of the sub-fin, followed by the stacks of low Ge% in SiGe/Si. The % of Ge in the regular SiGe layers is reduced to increase the selectivity between the regular SiGe layers and the bottom SiGe. However, due to the decrease in Ge% in the regular SiGe layers, the selectivity between the regular SiGe and the Si layers is reduced, thereby increasing the challenges of inner spacer formation and channel release [15]. This requires a process with high selectivity to prevent the etching of Si layers, which can lead to the formation of non-ideal inner spacers and channels, and may even cause yield issues [29].

Apart from the two additional steps not used in conventional structures in (a), all other steps are the same as those for conventional NSFET structures before the inner spacer formation. Subsequently, the bottom SiGe layers are etched (d), and the SiC layers are deposited (e). Fortunately, using plasma-based deposition techniques, researchers have developed many strategies to synthesize SiC thin films on silicon. As early as 1983, Shigehiro et al. proposed a reproducible process for producing single-crystal SiC with an intermediate buffer layer of sputtered SiC [30]. Furthermore, Nierlly et al. reported that the lattice mismatch problem between SiC and Si could be overcome by using an aluminum nitride (AlN) intermediate layer [31]. Therefore, it is desirable for SiC thin films to be grown on Si-substrates.

The remaining steps are identical to those in the conventional NSFET structure process, including the S/D epitaxy, dummy gate removal, channel release, gate oxide deposition, and metal gate formation. Therefore, compared to the process for the conventional NSFET structure, only four additional steps marked in red are required to achieve the SiC-NSFET structure.

### 2.3. Simulation Settings

The channel had a rectangular cross-sectional shape with rounded corners in this calibration work, and the crescent inner spacer was also designed [8]. The density gradient quantization model was used to consider the quantum confinement effect of the nanosheets. The bandgap narrowing Slotboom model was used to calculate the effective bandgap width, determining the intrinsic density. The recombination models included Shockley–Read–Hall (SRH), Auger, and Hurkx BTBT. As the thickness of the nanosheet channel was only a few nanometers ($N_{ch}$ = 5 nm), the mobility could not be expressed with a typical field-dependent interface model. Thus, the thin-layer mobility and Lombardi models were applied to account for the phonon and surface roughness scattering. The doping dependence model was specified to reflect the carrier impurity scattering. The high field saturation model was also included to describe the carrier velocity saturation effect at high electric fields. The thermodynamic model was used to simulate the effect of SHE on lattice temperature. The distributed interface thermal conductivity between the Si channel and ${\mathrm{SiO}}_{2}$ was also considered, with a value of 2 × $10^{-4}$ ${\mathrm{cm}}^{2}$/KW [32]. The other thermal parameters are listed in Table 2.

**Figure 3:**
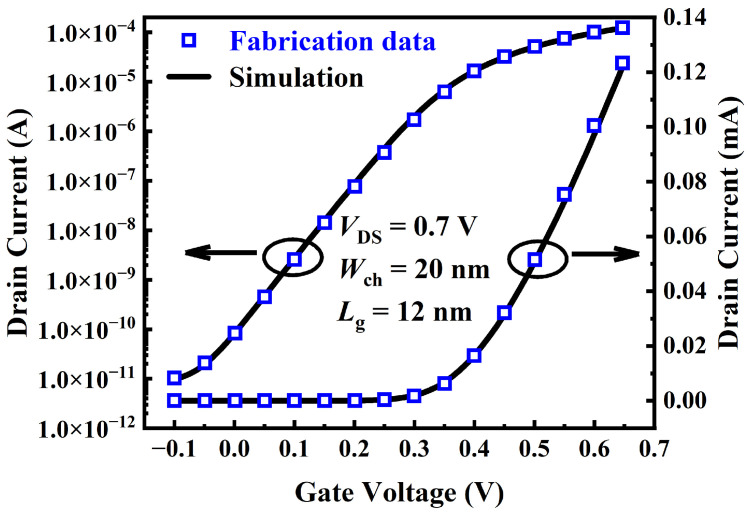
The calibration of transfer characteristics ($I_{d}$–$V_{g}$) of the conventional NSFET structure with experimental data from [8].

To ensure the accuracy of the following simulations, the physical parameters of NSFET were calibrated using the experimental data in [8]. Figure 3 shows good calibration with the experimental data. Under the negative bias, we calibrated the generation and recombination parameter values of the tunneling model. Under the positive bias, we adjusted the channel doping concentration and the gate metal work function to match the experimental results in the subthreshold region. Then, we adjusted the high-field saturation model parameters to make the simulation results match the experimental data in the saturation region.

## 3. Results and Discussion

### 3.1. Reduction in Leakage Current

In conventional punch-through stopper (PTS) substrates, the negative bias-driven band-to-band (BTBT) tunneling effect is a problem that needs to be solved [26]. The SiC-NSFET structure proposed in this work may be a potential solution. This section focuses on analyzing the leakage current of the SiC-NSFET and conventional structure under negative bias, highlighting the advantages of SiC-NSFET.

**Figure 4:**
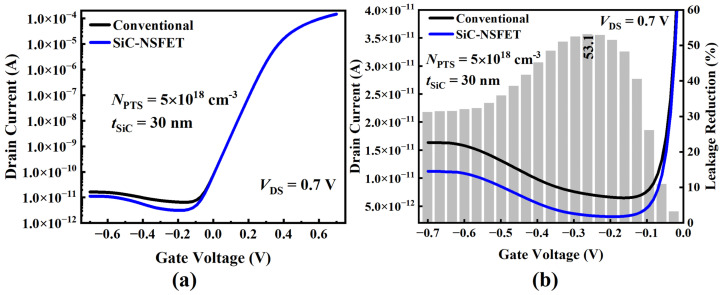
The transfer characteristics of the simulated NSFETs. (**a**) $I_{DS}$–$V_{GS}$ curves of the proposed SiC-NSFET and the conventional structure. (**b**) Drain current and leakage reduction of the two devices under negative gate bias.

Figure 4a illustrates the transfer characteristics of SiC-NSFET and a conventional structure. Under positive bias, it can be seen that the I–V curves of the two structures exhibit minimal differences with no significant variance. However, under the negative bias, the leakage current of SiC-NSFET is significantly lower than that of a conventional structure. Figure 4b plots the comparative analysis results of the transfer characteristics under negative bias to further analyze the difference in the leakage current between the two distinct structures. To show the leakage current suppression of the SiC-NSFET structure more intuitively, the gray bar graph in Figure 4b shows the SiC-NSFET leakage reduction compared to the conventional structure. The leakage reduction can be calculated from the following equation:

(1) $$Leakage\;reduction=\frac{I_{DS,Conv}-I_{DS,SiC}}{I_{DS,Conv}}$$

Here, $I_{DS,Conv}$ and $I_{DS,SiC}$ represent the leakage currents of the conventional structure and SiC-NSFET, respectively. Under $V_{GS}$ = 0 V and $V_{GS}$ = −0.7 V, the leakage current of the SiC-NSFET structure is decreased by 3.1% and 31.2%, respectively, compared with the conventional structure. The maximum leakage reduction is 53.1% under $V_{GS}$ = −0.25 V.

**Figure 5:**
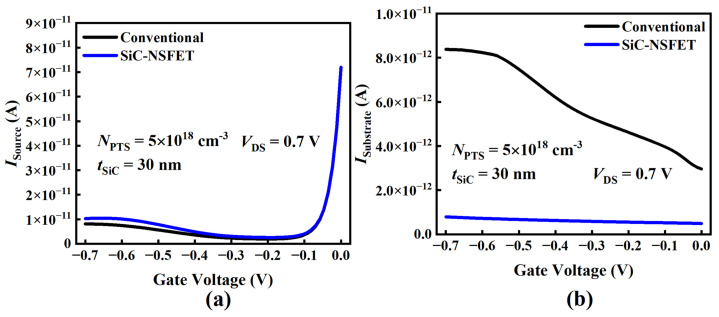
Comparison between different current components. (**a**) Source current, (**b**) substrate current under various negative gate voltages for SiC-NSFET and the conventional structure.

To analyze the leakage current suppression mechanism of the SiC-NSFET structure under negative bias, we divide the total drain leakage current ($I_{Drain}$) into two separate components. One component is the substrate leakage current ($I_{Substrate}$) caused by the minority carrier tunneling from the drain to the substrate. The other component is the drain-to-source leakage current ($I_{Source}$). Consequently, the total I drain can be expressed as the sum of $I_{Substrate}$ plus $I_{Source}$ [14,33]. Figure 5a illustrates the behavior of the $I_{Source}$ for both structures under negative bias. As the negative bias increases, $I_{Source}$ first decreases and then increases. Compared to the traditional structure, the $I_{Source}$ of the SiC-NSFET is slightly higher than that of the conventional structure. In contrast, the SiC-NSFET structure has apparent advantages in suppressing the substrate current, as shown in Figure 5b. Compared with the conventional structure, the $I_{Substrate}$ of SiC-NSFET is reduced by 89% on average, and the maximum reduction is 91.3%.

**Figure 6:**
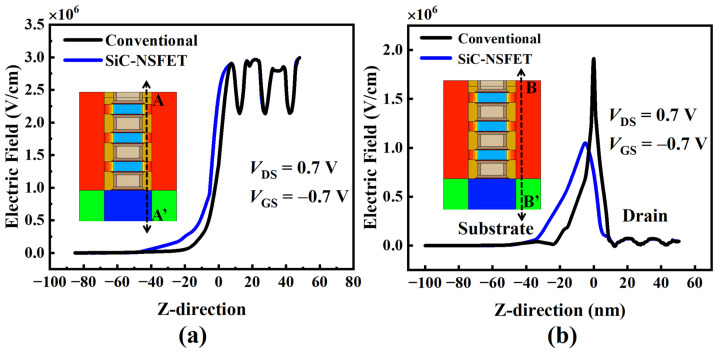
The electric field distribution along the z-axis for the above two NSFETs under negative bias at $V_{GS}$ = −0.7 V. (**a**) Across the substrate under the gate. (**b**) Across the substrate under the S/D.

**Figure 7:**
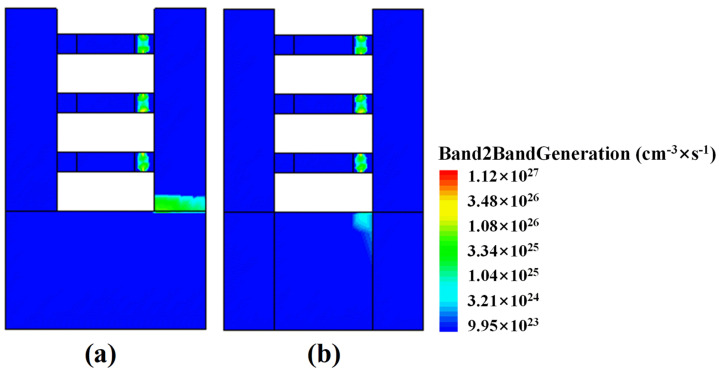
Cross-sectional view of BTBT generation distribution in (**a**) the conventional structure, and (**b**) SiC-NSFET under negative bias at $V_{GS}$ = −0.7 V.

The results in Figure 5 can be explained by Figure 6 and Figure 7. Due to SiC layers in the substrate of SiC-NSFET, there is a difference in the electric field. Figure 6a,b illustrate the electric field distribution of SiC-NSFET and conventional NSFET at the cutlines, A-$A^{\prime }$ and B-$B^{\prime }$, respectively. In the SiC-NSFET substrate, the electric field exhibits a more uniform distribution across the SiC layers, resulting in more electric field lines extending to the substrate under the gate. Therefore, compared with the conventional NSFET structure, the proposed SiC-NSFET has a weakened electric field under the drain-substrate junction and an enhanced electric field under the substrate beneath the gate. The larger the electric field, the higher the BTBT generation rate [34]. Therefore, compared with the conventional structure, the BTBT generation rate of SiC-NSFET in the substrate is higher at the cutline A-$A^{\prime }$. However, at the cutline, B-$B^{\prime }$, the BTBT generation rate at the drain-substrate junction decreases significantly, as shown in Figure 7. The higher the BTBT generation rate, the larger the BTBT current [33]. Therefore, SiC-NSFET exhibits a larger $I_{Source}$ and a smaller $I_{Substrate}$ than conventional devices.

**Figure 8:**
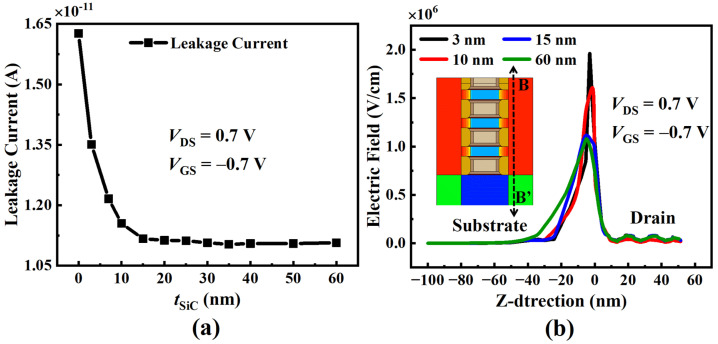
Simulation results of the above two NSFETs under negative bias at $V_{GS}$ = −0.7 V with different $t_{SiC}$ values. (**a**) Leakage current versus $t_{SiC}$ and (**b**) electric field distribution along the z-axis.

Figure 8a depicts the leakage current of the SiC-NSFET structure with varying SiC layer thicknesses. It can be seen from the figure that when the thickness is less than 15 nm, the leakage current increases as the thickness decreases. This is because a thin SiC layer cannot effectively disperse the electric field at the drain-substrate junction, resulting in a high $I_{Substrate}$. However, when the thickness of the SiC layer exceeds or equals 15 nm, the SiC layer significantly disperses the electric field at the drain-substrate junction, resulting in a reduction in $I_{Substrate}$ that is much greater than the increase in $I_{Source}$. Consequently, the leakage current decreases. Figure 8b exhibits the electric field distribution along the z-axis for SiC layers with different thicknesses. It can be seen that the peak electric field at the drain-substrate junction decreases as the SiC layer thickness increases. The electric field at the drain-substrate junction decreases significantly when the SiC layer thickness exceeds or equals 15 nm. Thus, to effectively suppress the substrate current, SiC-NSFET requires a SiC layer with a thickness of at least 15 nm.

**Figure 9:**
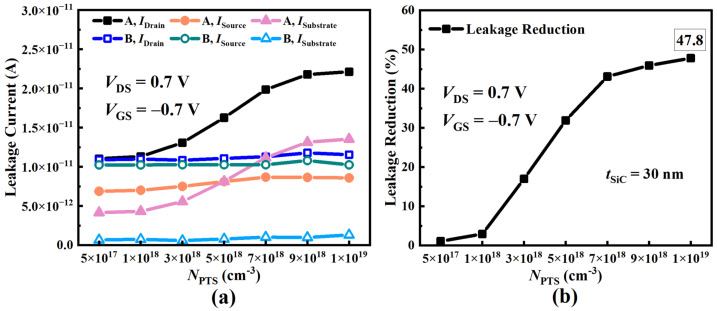
Simulation results of the above two NSFETs under negative bias at $V_{GS}$ = −0.7 V with different $N_{PTS}$ values. (**a**) Different current components and (**b**) leakage reduction versus $N_{PTS}$.

Figure 9a illustrates the leakage current variations in two structures with the $N_{PTS}$ at $T_{SiC}$ = 30 nm. The conventional structure is defined as device A, while the SiC-NSFET structure is defined as device B. As the $N_{PTS}$ increases, the leakage current in the conventional structure rises significantly, with the maximum value being 101.0% higher than the minimum. However, the leakage current in the SiC-NSFET remains relatively unchanged, with the maximum value only being 8.6% higher than the minimum. Because SiC layers scatter the drain-substrate junction electric field, the SiC-NSFET has a smaller drain-substrate junction electric field but a higher fringing electric field near SiC layers, resulting in a larger $I_{Source}$ and a lower $I_{Substrate}$. Typically, the $I_{Substrate}$ is much smaller than the $I_{Source}$. However, when the $N_{PTS}$ exceeds 5 × $10^{18}$ ${\mathrm{cm}}^{-3}$, the $I_{Substrate}$ in the conventional structure starts to surpass the $I_{Source}$ and even exceeds the total leakage current of the SiC-NSFET, becoming the dominant component of the leakage current. As the $N_{PTS}$ increases, the SiC-NSFET exhibits an enhanced suppression of the leakage current due to the distinct $I_{Substrate}$ behavior in the two structures, as shown in Figure 9b. When the $N_{PTS}$ is 1 × $10^{19}$ ${\mathrm{cm}}^{-3}$, the performance of SiC-NSFET is improved by 47.8% compared with the conventional structure. The range of reasonable $N_{PTS}$ for effectively suppressing the leakage current in the SiC-NSFET is much broader than in the conventional structure. Therefore, the SiC-NSFET demonstrates significantly better leakage current suppression than the conventional structure, especially at higher $N_{PTS}$.

### 3.2. Improvement in Thermal Reliability

**Figure 10:**
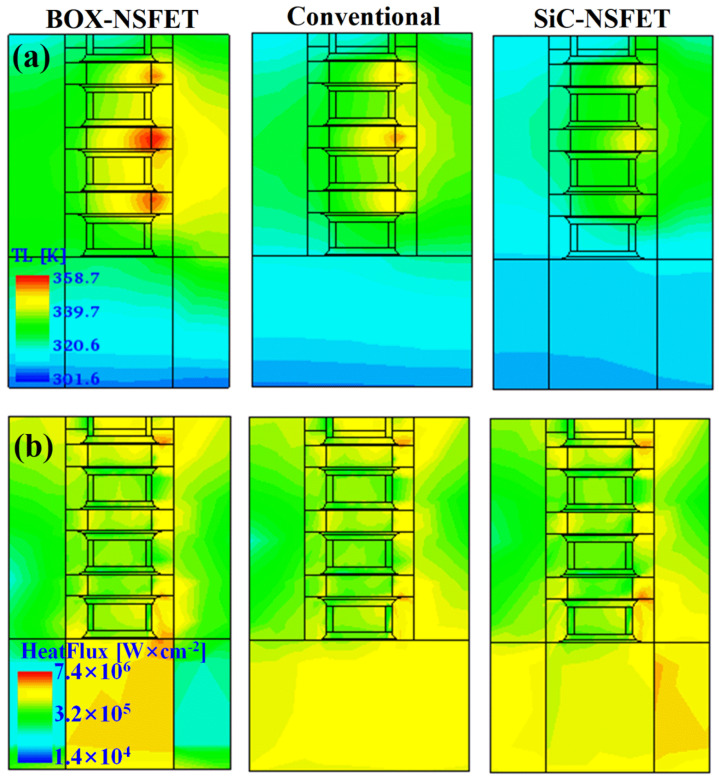
Heat distribution under $V_{GS}$ = $V_{DS}$ = 0.7 V in BOX-NSFET, conventional structure and SiC-NSFET. (**a**) Lattice temperature and (**b**) heat flux.

In this section, we analyze the SHE influence on the electrical characteristics of SiC-NSFET compared to the conventional structure and the buried oxide isolation NSFET (BOX-NSFET). The high-energy electrons collide with the lattice and lose energy, which is then transferred to the lattice, increasing lattice temperature ($T_{L}$) [35]. Figure 10 presents the distribution of $T_{L}$ and the heat flux for the three structures. Figure 10a shows that the BOX-NSFET has the highest $T_{L}$, while SiC-NSFET has the lowest. The maximum lattice temperature ($T_{L,max}$) is in the channel region, close to the drain extension, where the high electric field enhances the scattering between electrons and phonons. Figure 10b reveals that the heat flux is higher in the drain extension and its adjacent spacers compared to the source extension and its adjacent spacers. This is because the heat generated in the channel mainly accumulates near the drain extension, with the device primarily dissipating heat through the drain electrode [20]. At the same time, as the thermal conductivity of the material under S/D increases, the heat flux from CH2 to the substrate increases. In the BOX-NSFET, the significantly lower thermal conductivity of ${\mathrm{SiO}}_{2}$ compared to Si results in heat flux to the substrate being concentrated in the bulk region under the gate. However, in SiC-NSFET, the higher thermal conductivity of SiC leads to the heat flux primarily concentrated in the SiC layers under the S/D. Consequently, compared to the other two structures, the SiC-NSFET mitigates the SHE.

**Figure 11:**
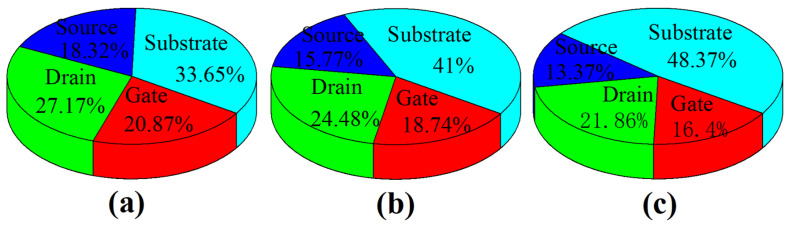
Heat flux in electrodes in (**a**) BOX-NSFET, (**b**) conventional structure, and (**c**) SiC-NSFET.

**Figure 12:**
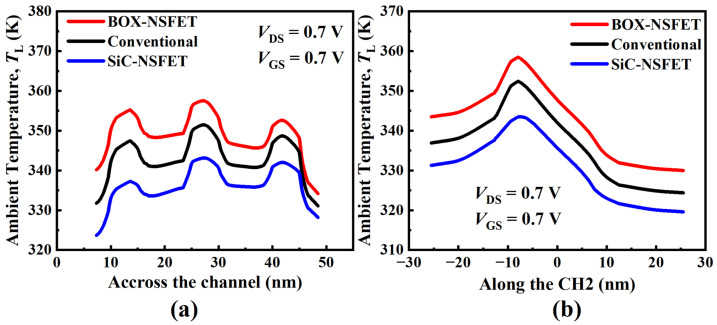
Cutline plot of lattice temperature (**a**) across the channels and (**b**) along the CH2.

Figure 11 shows the results of the heat flux ratio for the different electrodes in the three structures. From the results in Figure 11, a lot of heat flows toward the substrate in all three structures. When the thermal conductivity of the material under the S/D increases, the heat flow toward the substrate increases, leading to an increase in the heat flux ratio of the substrate electrodes. The heat flux ratios for the substrate electrode in the BOX-NSFET, conventional structure, and SiC-NSFET are 33.7%, 41.0%, and 48.4%, respectively. The device variation $T_{L}$ across the channel is presented in Figure 12a. It can be seen that the $T_{L}$ of CH2 is the highest due to its greater distance from the electrodes, resulting in a longer heat transfer path. Due to the difference in the heat flux ratio from the channel to the substrate, the $T_{L}$ of CH3 is higher than that of CH2 in BOX-NSFET and conventional structure. However, the $T_{L}$ of CH3 is lower than that of CH1 in SiC-NSFET. In addition, the $T_{L}$ differences between the nanosheets in the three structures are different. Figure 12b gives the variation of the $T_{L}$ device along the CH2. It can be seen that the maximum gradient of $T_{L}$ is in the channel region, compared to the S/D regions.

**Figure 13:**
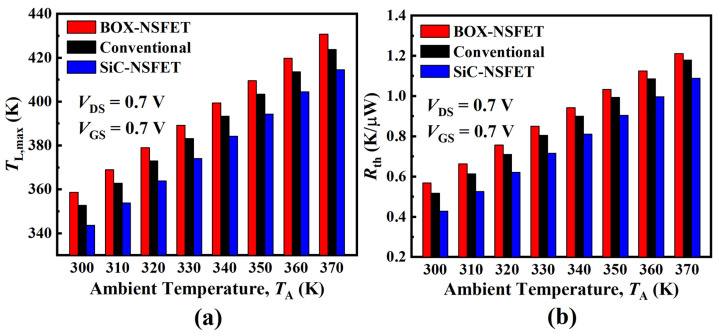
Variation of (**a**) maximum lattice temperature ($T_{L,max}$) and (**b**) effective thermal resistance versus ambient temperature.

Figure 13 shows the impact of ambient temperature ($T_{A}$) on the TL and effective thermal resistance ($R_{th}$ = ($T_{L}-T_{A}$)/$P_{in}$) of the three structures. As $T_{A}$ increases, both $T_{L,max}$ and $R_{th}$ of the device increase. When $T_{A}$ increases from 300 K to 370 K, $T_{L}$ and $R_{th}$ of the BOX-NSFET degrade by 20.1% and 113.6%, respectively. However, in SiC-NSFET, the thermal degradation caused by SHE can be alleviated as $T_{L}$ and $R_{th}$ are improved by 22.5% and 19.3%, respectively, compared with BOX-NSFET.

**Figure 14:**
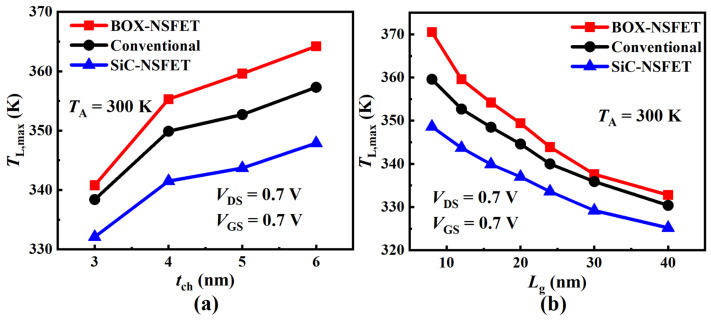
Impact of varying (**a**) the channel length and (**b**) the nanosheet thickness on the maximum lattice temperature ($T_{L,max}$) of the BOX-NSFET, SiC-NSFET, and the conventional structure.

In the nanoscale regime, the channel thickness ($t_{ch}$) and the gate length ($L_{g}$) significantly impact the thermal characteristics of NSFETs [36]. Therefore, we investigated the impact of different $t_{ch}$ and $L_{g}$ values on NSFETs. Figure 14a illustrates how $T_{L,max}$ varies with $t_{ch}$. As $t_{ch}$ increases, the channel volume increases, increasing the channel current [37]. In SiC-NSFET, the effect of SHE on the active regions is mitigated because the heat flows more to the substrate. When $t_{ch}$ increases, the $T_{L,max}$ of SiC-NSFET is significantly lower than that of BOX-NSFET and the conventional structure. Figure 14b explores how $T_{L,max}$ varies with $L_{g}$. As $L_{g}$ increases, the channel current decreases. The decreased electric field decreases the energy the carriers obtain in the drain extension region, reducing the scattering between the electrons and phonons. This results in a lower $T_{L,max}$. As $L_{g}$ increases, the heat flow to the gate electrode increases, decreasing the $T_{L,max}$ difference between different structures. However, the $T_{L,max}$ of SiC-NSFET is significantly reduced compared to BOX-NSFET and the conventional structure. In summary, the thermal characteristics of NSFETs can be optimized by adjusting the $t_{ch}$ and $L_{g}$ of the nanosheets.

## 4. Conclusions

In this work, we propose a SiC-NSFET structure that uses a PTS scheme only under the gate and SiC layers under the source and drain to improve the leakage current and thermal reliability. The I–V characteristics of NSFETs under negative bias were analyzed by separating the total leakage current into the source leakage current ($I_{Source}$) and substrate leakage current ($I_{Substrate}$). The results show that SiC-NSFET can effectively suppress the $I_{Substrate}$, and the performance improvement is up to 91.3% compared with the conventional structure. The optimal thickness of the SiC layer was found to be 15 nm. The SiC-NSFET exhibits superior immunity to punch-through stopper doping concentration ($N_{PTS}$) variations compared to the conventional structure. As the $N_{PTS}$ increases, the leakage current in the conventional structure degrades by 101.0%, while the SiC-NSFET exhibits minimal leakage current changes. At an $N_{PTS}$ of 1 × $10^{19}$ ${\mathrm{cm}}^{-3}$, the performance of SiC-NSFET is improved by 47.8% compared with the traditional structure. We also analyzed the self-heating effects (SHEs) of NSFETs under positive bias. The results indicate that the SiC-NSFET mitigates performance degradation due to the SHE, achieving the lowest lattice temperature ($T_{L,max}$) and thermal resistance ($R_{th}$). The majority of heat generated in the channel is dissipated to the substrate. The heat flux ratios of the substrate electrode for the BOX-NSFET, conventional structure, and SiC-NSFET are 33.7%, 41.0%, and 48.4%, respectively. As the ambient temperature ($T_{A}$) increases, both $T_{L,max}$ and $R_{th}$ increase. When $T_{A}$ rises from 300 K to 370 K, the SiC-NSFET effectively mitigates SHE as the $T_{L,max}$ and $R_{th}$ are improved by 22.5% and 19.3%, respectively, compared with BOX-NSFET. The SiC-NSFET achieves the lowest $T_{L,max}$ under varying the $t_{ch}$ and $L_{g}$. Therefore, the proposed SiC-NSFET structure significantly reduces the leakage current and improves thermal reliability, offering valuable insights for the further scaling of device sizes.

## Figures and Tables

**Table 1:** Geometry parameters for NSFETs.

Parameters	Values
Gate length, $L_{g}$	12 nm
Spacer length, $L_{sp}$	5 nm
Source/drain length, $L_{sd}$	13 nm
Contact gate pitch, CGP	48 nm
Channel width, $W_{ch}$	20 nm
Channel thickness, $t_{ch}$	5 nm
Thickness of SiC layers, $t_{SiC}$	30 nm
Vertical channel space, $N_{ch}$	10 nm
Equivalent oxide thickness, EOT	0.7 nm
Channel doping, $N_{channel}$	$1\times 10^{17}$ ${\mathrm{cm}}^{-3}$
Source/drain doping, $N_{SD}$	$1\times 10^{20}$ ${\mathrm{cm}}^{-3}$
Punch-through stopper (PTS) doping, $N_{PTS}$	$5\times 10^{18}$ ${\mathrm{cm}}^{-3}$
Contact resistance	$1\times 10^{-9}$ Ω·${\mathrm{cm}}^{2}$

**Table 2:** Thermal parameters for NSFETs.

Parameters	Values
Channel thermal conductivity, $K_{ch}$	8.07 W/(m·K)
Source/drain thermal conductivity, $K_{SD}$	16.61 W/(m·K)
Oxide thermal conductivity (${\mathrm{SiO}}_{2}$), $K_{SiO_{2}}$	1.4 W/(m·K)
High-k thermal conductivity (${\mathrm{HfO}}_{2}$), $K_{HfO_{2}}$	2.3 W/(m·K)
Inner spacer thermal conductivity, $K_{Si_{3}N_{4}}$	18.5 W/(m·K)
PTS thermal conductivity, $K_{PTS}$	148 W/(m·K)
Substrate thermal conductivity, $K_{sub}$	148 W/(m·K)

## Data Availability

All data that support the findings of this study are included within the article.
